# Supplementary material for: The Role of Standardized Phase Angle in the Assessment of Nutritional Status and Clinical Outcomes in Cancer Patients: A Systematic Review of the Literature
Source: Nutrients. 2022 Dec 22;15(1):50. doi: 10.3390/nu15010050 (PMC9824322; doi:10.3390/nu15010050)
Supplement: Supplementary file 1 [file nutrients-15-00050-s001.zip › nutrients-2044012-supplementary.pdf]

Supplementary Table S1. Search Strategy Details performed at April 17th, 2022.

Step	Search Strategy	Results
1. **Pubmed**		
#1	"Phase angle"[All Fields]	2,766
#2	"Neoplasms"[All Fields] OR "Neoplasia"[All Fields] OR "Neoplasias"[All Fields] OR "Neoplasm"[All Fields] OR "Tumors"[All Fields] OR "Tumor"[All Fields] OR "Cancer"[All Fields] OR "Cancers"[All Fields] OR "Malignancy"[All Fields] OR "Malignancies"[All Fields] OR "Malignant Neoplasms"[All Fields] OR "Malignant Neoplasm"[All Fields] OR "Neoplasm, Malignant"[All Fields] OR "Neoplasms, Malignant"[All Fields] OR "Benign Neoplasms"[All Fields] OR "Neoplasms, Benign"[All Fields] OR "Benign Neoplasm"[All Fields] OR "Neoplasm, Benign"[All Fields]	5,258,557
#3	#1 AND #2	249
2. **Embase**		
#1	'phase angle'/exp OR 'phase angle'	3,806
#2	'neoplasms'/exp OR neoplasms OR 'neoplasia'/exp OR neoplasia OR neoplasias OR 'neoplasm'/exp OR neoplasm OR 'tumors'/exp OR tumors OR 'tumor'/exp OR tumor OR 'cancer'/exp OR cancer OR 'cancers'/exp OR cancers OR 'malignancy'/exp OR malignancy OR 'malignancies'/exp OR malignancies OR 'malignant neoplasms' OR 'malignant neoplasm'/exp OR 'malignant neoplasm' OR 'neoplasm, malignant' OR 'neoplasms, malignant' OR 'benign neoplasms' OR 'neoplasms, benign' OR 'benign neoplasm'/exp OR 'benign neoplasm' OR 'neoplasm, benign'/exp OR 'neoplasm, benign'	7,305,563

#3	#1 AND #2	481
3. **Cochrane Library**		
#1	(“Phase Angle”):ti,ab,kw	261
#2	(Neoplasms):ti,ab,kw OR (Neoplasia):ti,ab,kw OR (Neoplasias):ti,ab,kw OR (Neoplasm):ti,ab,kw OR (Tumors):ti,ab,kw OR (Tumor):ti,ab,kw OR (Cancer):ti,ab,kw OR (Cancers):ti,ab,kw OR (Malignancy):ti,ab,kw OR (Malignancies):ti,ab,kw OR ("Malignant Neoplasms"):ti,ab,kw OR ("Malignant Neoplasm"):ti,ab,kw OR ("Neoplasm, Malignant"):ti,ab,kw OR ("Neoplasms, Malignant"):ti,ab,kw OR ("Benign Neoplasms"):ti,ab,kw OR ("Neoplasms, Benign"):ti,ab,kw OR ("Benign Neoplasm"):ti,ab,kw OR ("Neoplasm, Benign"):ti,ab,kw	231,749
#3	#1 AND #2	58
4. **Medline**		
#1	TS=(“Phase Angle”)	2,763
#2	TS=(Neoplasms) OR TS=(Neoplasia) OR TS=(Neoplasias) OR TS=(Neoplasm) OR TS=(Tumors) OR TS=(Tumor) OR TS=(Cancer) OR TS=(Cancers) OR TS=(Malignancy) OR TS=(Malignancies) OR TS=("Malignant Neoplasms") OR TS=("Malignant Neoplasm") OR TS=("Neoplasm, Malignant") OR TS=("Neoplasms, Malignant") OR TS=("Benign Neoplasms") OR TS=("Neoplasms, Benign") OR TS=("Benign Neoplasm") OR TS=("Neoplasm, Benign")	4,429,992
#3	#1 AND #2	226
5. **Web of Science**		
#1	TS=(“Phase Angle”)	11,007

#2	TS=(Neoplasms) OR TS=(Neoplasia) OR TS=(Neoplasias) OR TS=(Neoplasm) OR TS=(Tumors) OR TS=(Tumor) OR TS=(Cancer) OR TS=(Cancers) OR TS=(Malignancy) OR TS=(Malignancies) OR TS=("Malignant Neoplasms") OR TS=("Malignant Neoplasm") OR TS=("Neoplasm, Malignant") OR TS=("Neoplasms, Malignant") OR TS=("Benign Neoplasms") OR TS=("Neoplasms, Benign") OR TS=("Benign Neoplasm") OR TS=("Neoplasm, Benign")	3,412,398
#3	#1 AND #2	352
6. **CINAHL**		
#1	SU=("Phase Angle")	334
#2	SU=(Neoplasms) OR SU=(Neoplasia) OR SU=(Neoplasias) OR SU=(Neoplasm) OR SU=(Tumors) OR SU=(Tumor) OR SU=(Cancer) OR SU=(Cancers) OR SU=(Malignancy) OR SU=(Malignancies) OR SU=("Malignant Neoplasms") OR SU=("Malignant Neoplasm") OR SU=("Neoplasm, Malignant") OR SU=("Neoplasms, Malignant") OR SU=("Benign Neoplasms") OR SU=("Neoplasms, Benign") OR SU=("Benign Neoplasm") OR SU=("Neoplasm, Benign")	2,312,346
#3	#1 AND #2	110

Supplementary Table S2. Quality assessments of included studies using by MMAT.

Study	Screening Questions		Quantitative Non-Randomized Studies					Total	Classification
	S1	S2	1	2	3	4	5		
Pena *et al.*[13], 2019	*	*	*	*	*	*	*	*****	100%
Axelsson *et al.*[45], 2018	*	*	*	*	*	*	*	*****	100%
Urbain *et al.*[49], 2013	*	*	*	*	*	*	*	*****	100%
Norman *et al.*[34], 2010	*	*	*	*	*	*	*	*****	100%
Maurício *et al.*[50], 2018	*	*	*	*	*	*	*	*****	100%
Leon-Idougourram *et al.*[51], 2022	*	*	*	*	*	-	*	****	80%
Harter *et al.*[52], 2017	*	*	*	*	*	-	*	****	80%
Hui *et al.*[46], 2014	*	*	*	*	*	*	*	*****	100%
Roccamatisi *et al.*[40], 2021	*	*	*	*	*	*	*	*****	100%
Yates *et al.*[47], 2020	*	*	*	*	-	*	*	****	80%
Paiva *et al.*[44], 2011	*	*	*	*	*	*	*	*****	100%
Cereda *et al.*[53], 2021	*	*	*	*	*	*	*	*****	100%
Paixao *et al.*[48], 2021	*	*	*	*	*	*	*	*****	100%

Abbreviation: S1: Are there clear research questions?; S2: Do the collected data allow to address the research questions?; 1: Are the participants representative of the target population?; 2: Are measurements appropriate regarding both the outcome and intervention (or exposure)? 3: Are there complete outcome data?; 4: Are the confounders accounted for in the design and analysis?; 5: During the study period, is the intervention administered (or exposure occurred) as intended? *: Yes; -: No; /: Can't tell.

Supplementary Table S3. Mentioned outcomes related to SPA.

Study	Nutritional status	Clinical Outcomes	
		Complication	Survival
Pena *et al.*[13], 2019	*	*	*
Axelsson *et al.*[45], 2018			*
Urbain *et al.*[49], 2013			*
Norman *et al.*[34], 2010	*		*
Maurício *et al.*[50], 2018		*	
Leon-Idougourram *et al.*[51], 2022	*		
Harter *et al.*[52], 2017		*	
Hui *et al.*[46], 2014			*
Roccamatisi *et al.*[40], 2021		*	
Yates *et al.*[47], 2020	*		*
Paiva *et al.*[44], 2011			*
Cereda *et al.*[53], 2021	*		*
Paixao *et al.*[48], 2021			*

Abbreviation: *: mentioned.
